# Vomiting among Cattle

**Published:** 1896-09

**Authors:** M. Perrussel


VOMITING AMONG CATTLE (by M. Perrussel in *Le Progrès Vétérinaire*). The cow which the author reports had eaten in the evening some grass of a second crop which had just been harvested, then the next day some more of the grass mixed with clover, which had been cut for a week, with bran and with the leaves of beet-root. After the second meal, composed of green lucern (*Medicago sativa*), colicky symptoms appeared and a certain degree of dyspnœa, then at the end of some minutes a fit of coughing, eructations, and vomiting, which rid the animal of the lucern. After this was over rumination followed in a perfectly normal manner. The same occurrence took place again the evening afterward after a meal of lucern, and the following morning after eating hay. The author, when called to attend the cow, found everything normal barring a little distention of the rumen, and on giving her some hay witnessed the series of phenomena mentioned above. Vomiting was accomplished with considerable effort: the head, neck, and shoulders were stretched out, the back arched, and the legs drawn together.

The ingestion of liquids gave no occasion for a manifestation of this kind. The cause of these phenomena, according to the author, is found in the dilatation which the rumen had suffered by the ingestion of a large quantity of fresh grass, in the tardy rumination of the same (the animal had been yoked up and had had to pull a load just after stuffing itself with grass), and in the ulterior prehension of fermentable food. These unauthentic data led him to discard the hypothesis of orificial tumors of the stomach ; the absence of dysphagia argued against the existence of a foreign body in the œsophagus. In the reporter's opinion the dilatation could have been the consequence of a momentary over-feeding, but the dilatation did not seem to explain fully the periodic throwing up of the food; there must have been connected with it an exaggerated sensibility of the wall whose excitations caused the violent reflex actions. This view is further confirmed by the quick response to the medicine furnished, which was as follows: 10 grammes of camphor and 10 grammes of bromide of potassium, together with sulphate of sodium in the form of a mucilaginous drench. A very low diet for twenty-four hours, with an increase of food the following day completed the therapeutical means employed, and at the end of eight days the animal returned to its normal diet without inconvenience.—*Annales de Médecine Vétérinaire*, March, 1896.

